# Architecture of Class 1, 2, and 3 Integrons from Gram Negative Bacteria Recovered among Fruits and Vegetables

**DOI:** 10.3389/fmicb.2016.01400

**Published:** 2016-09-13

**Authors:** Daniela Jones-Dias, Vera Manageiro, Eugénia Ferreira, Paula Barreiro, Luís Vieira, Inês B. Moura, Manuela Caniça

**Affiliations:** ^1^National Reference Laboratory of Antibiotic Resistances and Healthcare Associated Infections, Department of Infectious Diseases, National Institute of Health Doutor Ricardo JorgeLisbon, Portugal; ^2^Centre for the Studies of Animal Science, Institute of Agrarian and Agri-Food Sciences and Technologies, Oporto UniversityOporto, Portugal; ^3^Innovation and Technology Unit, Human Genetics Department, National Institute of Health Doutor Ricardo JorgeLisbon, Portugal

**Keywords:** fresh produce, agriculture, antibiotic resistance, mobile genetic elements, integrons

## Abstract

The spread of antibiotic resistant bacteria throughout the food chain constitutes a public health concern. To understand the contribution of fresh produce in shaping antibiotic resistance bacteria and integron prevalence in the food chain, 333 antibiotic resistance Gram negative isolates were collected from organic and conventionally produced fruits (pears, apples, and strawberries) and vegetables (lettuces, tomatoes, and carrots). Although low levels of resistance have been detected, the bacterial genera identified in the assessed fresh produce are often described not only as environmental, but mostly as commensals and opportunistic pathogens. The genomic characterization of integron-harboring isolates revealed a high number of mobile genetic elements and clinically relevant antibiotic resistance genes, of which we highlight the presence of as *mcr-1, qnrA1, bla*_GES−11_, *mphA*, and *oqxAB*. The study of class 1 (*n* = 8), class 2 (*n* = 3) and class 3 (*n* = 1) integrons, harbored by species such as *Morganella morganii, Escherichia coli, Klebsiella pneumoniae*, led to the identification of different integron promoters (PcW, PcH1, PcS, and PcW_TNG−10_) and cassette arrays (containing *drfA, aadA, cmlA, estX, sat*, and *bla*_GES_). In fact, the diverse integron backbones were associated with transposable elements (e.g., Tn*402*, Tn*7, ISCR*1, Tn2^*^, IS*26*, IS*1326*, and IS*3*) that conferred greater mobility. This is also the first appearance of In*1258*, In*1259*, and In*3-13*, which should be monitored to prevent their establishment as successfully dispersed mobile resistance integrons. These results underscore the growing concern about the dissemination of acquired resistance genes by mobile elements in the food chain.

## Introduction

The spread of antimicrobial resistance has made bacterial infections gradually more difficult to treat (Blair et al., 2015; Hawkey, 2015). Increasing evidence suggests that intestinal microbiota of humans and animals constitutes a reservoir for antibiotic resistant bacteria and resistance genes (Hu et al., 2014). In fact, antibiotic resistant community- and hospital-acquired infections are often caused by bacteria that may inhabit the human gut (Cantas et al., 2013). Consequently, a better understanding on how antibiotic resistant bacteria break into the human microbiota is essential to prevent multidrug resistant infections.

Fresh produce frequently harbor non-pathogenic environmental microorganisms (Aserse et al., 2013). During growth and harvesting, vegetables and fruits can also become contaminated with pathogenic and commensal bacteria from animals and humans. This contamination can occur in the field through direct contact with soil, and through the application of manure and wastewater as biofertilizers (Ben Said et al., 2015; Berendonk et al., 2015; van Hoek et al., 2015). In addition, crops are subjected to high selection pressure caused by exposure to antibiotic residues indirectly coming from manure and wastewater, or directly from phytopharmaceutical agents (Gaze et al., 2011; Finley et al., 2013). Thus, fruits and vegetables that are grown close to the soil, and that are not subjected to any type of cooking process are especially prone to transmit contaminant microorganisms (Berger et al., 2010). However, it should be noted that fresh produce might also become contaminated in stores, after distribution, through incorrect human manipulation (van Hoek et al., 2015).

In Portugal, the majority of the fruits and vegetables that are available to the consumers are conventionally produced (GPP, 2011). However, organic produce constitute a viable alternative that is getting more supporters every year in western countries (GPP, 2011; Jensen et al., 2011). According with the European rules, although organic production does not comprise the use of chemical agents, it still allows the use of manure from farming and sewage (European Commission, 1991). The overall influence of conventional and organic produce in the exposure of consumers to resistant bacteria has been evaluated in France in 2009, showing that those contained equivalent amounts of antibiotic resistant Gram negative bacteria (Ruimy et al., 2010). However, a recent study from Portugal analyzing different agricultural soil samples, revealed that exposure to conventional agricultural practices constituted a risk factor for non-susceptibility to many antibiotics, multidrug resistance, and production of Extended-Spectrum β-Lactamases (ESBL; Jones-Dias et al., 2016).

Horizontal transfer of genetic material among Gram negative bacteria plays an important role in the dissemination of multidrug resistance. The location of antibiotic resistant genes on mobile genetic elements, such as plasmids and transposons, facilitates the horizontal mobilization of resistance among microorganisms. Integrons are also associated to the complex dynamics of antibiotic resistance (Stokes and Gillings, 2011). These latter elements are mainly comprised of an integrase gene (*intI*) whose product allows them to capture and collect gene cassettes through a recombination site (*attI*). In turn, the integron itself may be directly associated with transposable elements, which drive their mobility in the cell, such as transposon 402 (Tn*402*) in class 1 integrons and transposon 7 (Tn*7*) in class 2 integrons (Cambray et al., 2010; Stokes and Gillings, 2011). Classes 1, 2, and 3 integrons display elevated clinical importance, being in part responsible for the ongoing accumulation of genes cassettes coding for antibiotic resistance genes (Escudero et al., 2015). Recently, a study by Gillings et al. (2015) proposed the use of class 1 integrase-encoding gene as a generic marker for anthropogenic pollutants, due to its common association with antibiotic resistance and heavy metals, and its appearance in pathogenic and non-pathogenic bacteria (Gillings et al., 2015).

In this study, we aimed to understand the involvement of vegetable products in the transmission of antibiotic resistant and integron-harboring Gram negative bacteria. To address our aims, we examined the occurrence and diversity of integrons in Gram negative isolates collected from fresh fruits and vegetables, organically and conventionally produced in Portugal. The association of integrons with proper mobile genetic elements was also addressed in order to assess the putative dissemination potential.

## Materials and methods

### Fresh produce sampling

Between March 2013 and February 2014, one conventional and one organic batch of three fruits and three vegetables were purchased, once a month, as available (Table 1). Produce were grown and marketed in Portugal and acquired at retail stores located in the region of Lisbon and Tagus Valley. The specimens were meant to represent the most consumed fresh produce in Portugal. Organic products were either bought at certified specialized stores, or were differentiated from conventional produce by the presence of specific labels, indicating that the producer respects specific regulation. Overall, we evaluated 144 products, including 74 vegetables and 70 fruits. Of these, 24 were green leaf lettuces (*Lactuca sativa* var. *romana*), 26 tomatoes (*Solanum lycopersicum*), 24 carrots (*Daucus carota*), 20 pears (*Pyrus communis*), 26 apples (*Malus* spp.) and 24 were strawberries (*Fragaria* spp). The exact same number of samples was collected from conventional and organic products. All products were immediately transported to the laboratory and processed: 50 g of each product was selected at random without washing or peeling, diluted 1:5, homogenized (Stomacher 80 Biomaster®, Seward, UK), labeled, enriched 12 h at 37°C, and frozen at −80°C (Ruimy et al., 2010).

**Table 1:** **Distribution of 144 samples (70 fruits and 74 vegetables) according with date of collection, store, and produce**.

**Date of collection (Month/Year)**	**Conventional**		**Organic**	
	**Store code**	**Produce**	**Store code**	**Produce**
March, 2013	F	Ap, Cr, Le, Pe, St, To	C	Ap, Cr, Le, Pe, To
April, 2013	I	Ap, Cr, Le, Pe, St, To	E	Ap, Cr, Le, To, St
May, 2013	G	Ap, Cr, Le, St, To	C	Ap, Cr, Le, St, To
June, 2013	L	Ap, Cr, Le, St, To	K	Ap, Cr, Le, St, To
July, 2013	C	Ap, Pe, St, To	G	Cr, Le, Pe, To
August, 2013	A	Ap, Le, St, To	J	Ap, Cr, Le, St, Pe
September, 2013	L	Ap, Cr, Le, Pe, To	B	Ap, Le, Pe, To
October, 2013	H	Ap, Le, Pe, To	D	Ap, Cr, Le, Pe, To
November, 2013	G	Ap, Cr, Le, Pe, To, St	G	Ap, To
December, 2013	J	Ap, Le, Pe, To	A	Ap, Le, Pe, To
January, 2014	J	Cr, Pe, St	E	Cr, Le
February, 2014	J	Ap, Cr, St, To	C	Cr, Le, St

*Ap, Apple (n = 26); Cr, Carrot (n = 24); Le, Lettuce (n = 24); Pe, Pear (n = 20); St, Strawberry (n = 24); To, Tomato (n = 26)*.

### Selection and identification of antibiotic resistance bacteria

Portions of the resultant fluids were spread-plated onto violet red bile glucose agar (VRBG) plates each containing specific concentrations of different antibiotics: 100 mg/L of amoxicillin, 2 mg/L of cefotaxime, 2 mg/L of ceftazime, 4 mg/L of ertapenem, 2 mg/L of imipenem, 10 mg/L of tetracycline, 20 mg/L of chloramphenicol, 50 mg/L of nalidixic acid, 4 mg/L of ciprofloxacin or 2 mg/L of gentamicin, and incubated at 37°C for 18–24 h. Control plates without antibiotic and with each antibiotic were used to assess total recovery of each sample and the integrity of the antibiotic selection protocol. The selection of 333 resistant Gram negative bacteria (Table 1, Tables S1, S2) was performed as previously described (Jones-Dias et al., 2016). Briefly, colonies obtained with each commodity were grouped by color, texture and size prior to representative numbers of them being streaked onto fresh VRBG agar to obtain an array of discrete colonies. Bacteria displaying a persistent, homogenous morphotype were considered pure strains, as previously reported (Bezanson et al., 2008; Jones-Dias et al., 2016). Individual colonies were selected based on their morphology so that no putative duplications were included, and all perceivable morphologically distinguishable colony types were sampled.

The isolates were identified through the amplification of the 16S ribosomal RNA (rRNA) gene, as previously described (Jones-Dias et al., 2016). PCR products were then purified with ExoSAP IT (USB Corporation, Cleveland, OH), and further sequenced directly, on both strands, using automatic sequencer ABI3100 (Applied Biosystems, Warrington, UK). The resulting sequences were then analyzed using the Bionumerics software (Applied Maths, Sint-Martens-Latem, Belgium) and assigned to respective identification, using the tools available on the NCBI website (http://blast.ncbi.nlm.nih.gov/Blast.cgi).

### Antibiotic susceptibility testing

Among the 333 isolates selected, antibiotic susceptibility was analyzed for 320 isolates due to the inexistence of specific breakpoints to evaluate non-susceptibility results from *Aeromonas* spp., *Comamonas* spp., and *Delftia* spp. (*n* = 12). Isolates from genera *Stenotrophomonas* (*n* = 13) were only evaluated toward sulfamethoxazole/trimethoprim, which corresponds to the only breakpoint available for that bacterial species. The antibiotic susceptibility of remaining isolates (*n* = 308), distributed among *Enterobacteriaceae, Acinetobacter* spp. (family *Moraxellaceae*), and *Pseudomonas* spp. (family *Pseudomonadaceae*) was performed by disc diffusion method against the following antibiotics (Biorad): amoxicillin (25 μg), ampicillin (10 μg), cefotaxime (5 μg), ceftazidime (10 μg), cefepime (30 μg), cefoxitin (30 μg), ertapenem (10 μg), imipenem (10 μg), amoxicillin with clavulanic acid (20 + 10 μg), nalidixic acid (30 μg), ciprofloxacin (5 μg), gentamicin (15 μg), amikacin (30 μg), chloramphenicol (75 μg), tetracycline (30 μg), and trimethoprim with sulfamethoxazole (1.25 + 23.75 μg). Interpretation of results was performed according to the cut-off values recommended by the European Committee on Antimicrobial Susceptibility Testing (EUCAST, http://mic.eucast.org/Eucast2/); however, for *Acinetobacter* spp., four clinically relevant antibiotics were interpreted according to the Antibiogram Committee of the French Society of Microbiology (CA-SFM, http://www.sfm-microbiologie.org/) due to non-existence of EUCAST breakpoints (Table 2). Isolates were considered multidrug resistant when presenting non-susceptibility to three or more structurally unrelated classes of antibiotics (Magiorakos et al., 2011). Double disk synergy test was used to phenotypically detect the presence of specific groups of β-lactamases in the 333 isolates: amoxicillin (25 μg) and amoxicillin plus clavulanic acid (20 + 10 μg) for the phenotypic detection of penicillinases, cefotaxime (5 μg) and amoxicillin clavulanic acid (20 + 10 μg) for ESBL, cefoxitin (30 μg) and cloxacillin (750 μg) for AmpC β-lactamases, and imipenem (5 μg) and imipenem plus dipicolinic acid (750 μg) for metallo-β-lactamases. *Escherichia coli* ATCC® 25922™ was also tested as a control of this technique (Jones-Dias et al., 2014).

**Table 2:** **Percentage of isolates from organic and conventionally produced fruits and vegetables non-susceptible to antibiotics according with their family (*n* = 308)**.

**Antibiotic**	***Enterobacteriaceae (n* = 184)**		***Acinetobacter spp. (n* = 89)**		***Pseudomonas spp. (n* = 35)**	
	**Organic (*n* = 83)**	**Conventional (*n* = 101)**	**Organic (*n* = 30)**	**Conventional (*n* = 58)**	**Organic (*n* = 10)**	**Conventional (*n* = 25)**
**β-LACTAMS**						
Ampicillin	29.3	45.1	NA	NA	NA	NA
Cefotaxime	6.5	5.4	31.5^a^	63.0^a^	NA	NA
Ceftazidime	5.4	5.4	6.7^a^	13.5^a^	0	8.6
Cefepime	0.0	0.0	0^a^	0^a^	2.9	5.7
Cefoxitin	19.6	26.6	NA	NA	NA	NA
Imipenem	0.0	0.0	1.1	0	2.9	8.6
Ertapenem	2.2	1.1	NA	NA	NA	NA
**QUINOLONES**						
Nalidixic acid	3.3	4.9	NA	NA	NA	NA
Ciprofloxacin	1.1	0.0	1.1	0	0	0
**AMINOGLYCOSIDES**						
Gentamicin	1.1	3.8	1.1	0	2.9	0
Amikacin	0.5	0.5	1.1	0	2.9	0
**TETRACYCLINES**						
Tetracycline	NA	NA	1.1^a^	0^a^	NA	NA
**PHENICOLS**						
Chloramphenicol	3.8	3.8	NA	NA	NA	NA
**SULFONAMIDES**						
Trimethoprim/ Sulfamethoxazole	1.1	0.5	NA	NA	NA	NA

a *CA-SFM breakpoints were applied due to inexistence of EUCAST breakpoints; NA, No EUCAST or CA-SFM breakpoints were available for the antibiotic*.

### Molecular detection of class 1, 2, and 3 integrons

All isolates were investigated for the presence of class 1, 2, and 3 integrase-encoding genes, through PCR amplification using primers reported elsewhere (Leverstein-Van Hall et al., 2002; Corrêa et al., 2014; Manageiro et al., 2015).

### Whole genome sequencing of integron-carrying isolates

All integrase-positive isolates were characterized by whole genome sequencing. Briefly, genomic DNA was extracted using DNeasy Blood and Tissue Kit (Qiagen, Aarhus) and quantified using Qubit 1.0 Fluorometer (Invitrogen, Waltham). The Nextera XT DNA Sample Preparation Kit (Illumina, San Diego, CA) was used to prepare sequencing libraries from 1 ng of genomic DNA according to the manufacturer's instructions. Paired-end sequencing of 250 bp reads was performed on a MiSeq (Illumina). Analysis of the integrase-producing isolates was carried out as described elsewhere (Jones-Dias et al., 2015). Briefly, PathogenFinder 1.1, ResFinder 2.1 (90% identity and 40% minimum length), and PlasmidFinder 1.3 (< 98% homology) were used to estimate the number of pathogenicity determinants, antibiotic resistance genes and plasmids, respectively, within the genome (Zankari et al., 2012; Cosentino et al., 2013; Carattoli et al., 2014). The NCBI prokaryotic genome automatic annotation pipeline (PGAAP) was used for annotation (http://www.ncbi.nlm.nih.gov/genome/annotation_prok). Specific analysis of class 1, 2, and 3 integrons was also carried out with CLC genomics workbench version 8.5.1 (Qiagen). Contigs carrying integrons were manually assembled and annotated whenever was necessary. Isolates showing novel Multilocus Sequence Typing (MLST) alleles combinations were assigned to sequence types (STs) through the respective MLST websites (http://pubmlst.org/ecloacae/ and https://enterobase.warwick.ac.uk/).

### Nucleotide sequence accession numbers

This Whole Genome Shotgun project has been deposited at DDBJ/EMBL/GenBank under the accession PRJNA311932. The versions described in this paper are the following: LSUR00000000 (*Enterobacter cloacae* INSali2), PRJNA311932 (*E. cloacae* INSali10), LSRK00000000 (*E. coli* INSali25), LSUT00000000 (*E. coli* INSali38), LSUU00000000 (*E. coli* INSali92), LSUV00000000 (*Raoultella planticola* INSali127), LSUW00000000 (*R. planticola* INSali133), LSUX00000000 (*Morganella morganii* INSali207), LSUY00000000 (*E. coli* INSali370), LSUZ00000000 (*Pseudomonas putida* INSali382) and LSVA00000000 (*Klebsiella pneumoniae* INSali390).

### Integrons sequence submission

The integron sequences were submitted to the INTEGRALL database (http://integrall.bio.ua.pt) for annotation and integron number assignment.

## Results

### Gram negative population of fresh fruits and vegetables

Three hundred and thirty three Gram negative antibiotic resistant isolates, cultured from 72 conventionally and 72 organically produced fruits (*n* = 70) and vegetables (*n* = 74) were obtained upon selection with the antibiotics previously referred (Table 1, Tables S1, S2). Among the 144 samples from fresh produce, 78 samples carried Gram negative bacteria, among which, 39 were conventionally and 39 were organically produced. Globally, 195 and 138 bacterial isolates were recovered from fruits and vegetables conventionally and organically produced, respectively.

**Figure 1:**
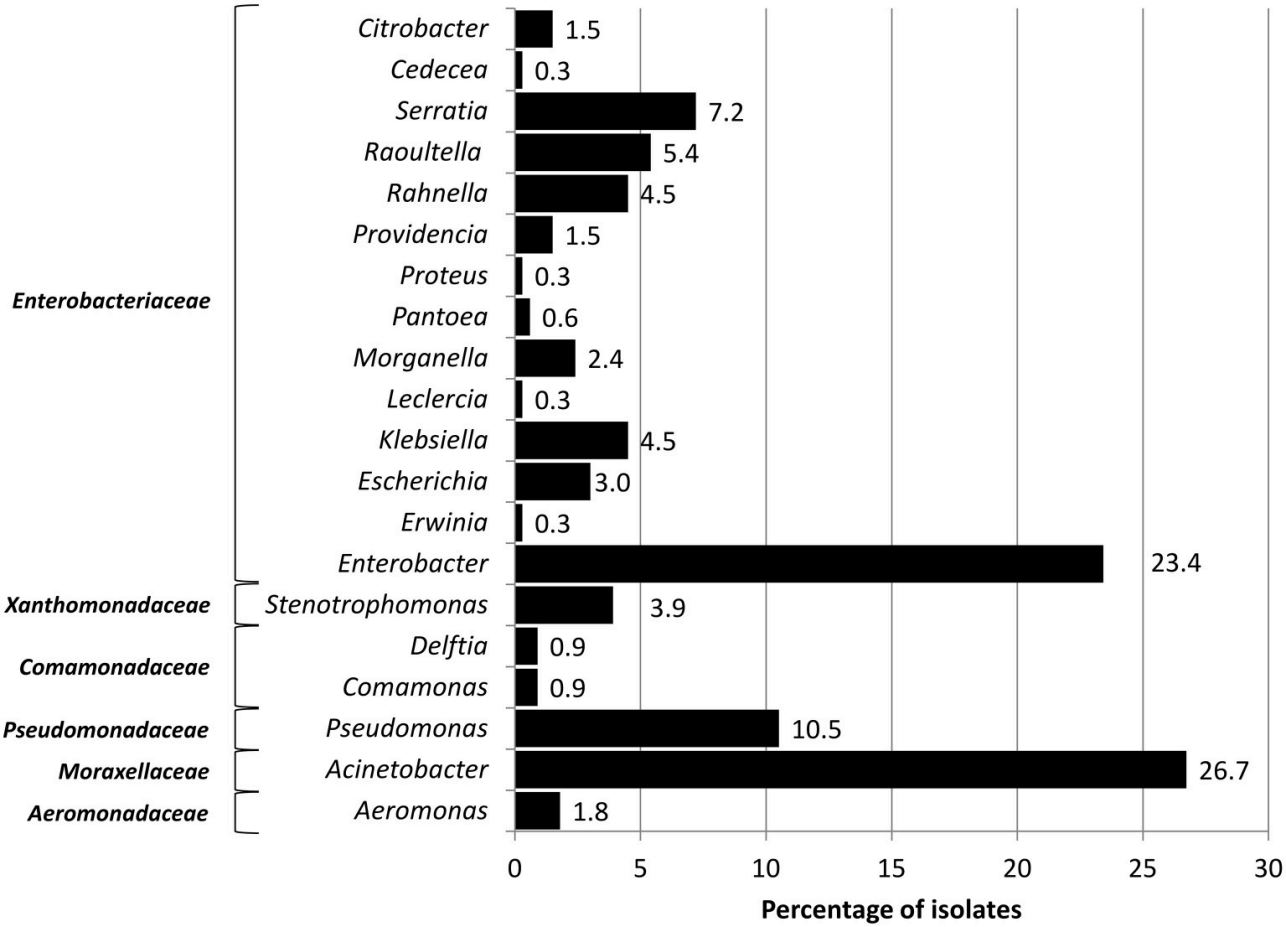
**Percentage of bacterial genera recovered from fruits and vegetables**.

16S rDNA profiling revealed that the isolates were represented by 20 bacterial genera distributed among six main families of Gram negative bacteria, among which the predominant was *Enterobacteriaceae* (55.3%; Figure 1). The remaining groups comprised *Moraxellaceae* (*Acinetobacter*, 26.7%), *Pseudomonadaceae* (*Pseudomonas*, 10.5%), *Xanthomonadaceae* (*Stenotrophomonas*, 3.9%), *Comamonadaceae* (1.8%) [*Delftia* (0.9%) and *Comamonas* (0.9%)], and *Aeromonadaceae* (*Aeromonas*, 1.8%; Figure 1).

### Evaluation of antibiotic susceptibility

For *Enterobacteriaceae* (*n* = 184), β-lactam non-susceptibility ranged between 0% for imipenem/cefepime and 45.1% for ampicillin, in isolates from conventionally produced fruits and vegetables (Table 2). Variations regarding β-lactam non-susceptibility were detected among the modes of produce production for *Enterobacteriaceae*: an average of 9% for organic and 11.9% for isolates recovered from conventionally produced items. Globally, values of non-susceptibility for the two quinolones tested ranged between 0% and 4.9% for conventional produce (Table 2). Non-susceptibility values for *Enterobacteriaceae* against aminoglycosides (0.5–3.8%), chloramphenicol (3.8%) and sulfonamides (0.5–1.1%) showed similar results between isolates recovered from organic and conventional fruits and vegetables (Table 2).

For *Acinetobacter* spp. (*n* = 89), non-susceptibility to β-lactam antibiotics ranged between imipenem/cefepime (0%) for both types of production, and cefotaxime (63.0%) for isolates recovered from conventionally produced fruits and vegetables. Non-susceptibility to other assessed antibiotics (ciprofloxacin, gentamicin, amikacin, and tetracycline) showed 0–1.1% of non-susceptibility in bacteria from this genus recovered from conventional and organic production, respectively (Table 2).

When *Pseudomonas* spp. (*n* = 35) isolates were analyzed, the minimum values of antibiotic non-susceptibility fluctuated between 0% for ceftazidime/ciprofloxacin and ciprofloxacin/gentamicin/amikacin for organic and conventional produce, respectively. Maximum non-susceptibility values corresponded to 8.6% for ceftazidime/imipenem for isolates recovered from conventionally produced items (Table 2).

*Stenotrophomonas malthophilia* isolates (*n* = 13) recovered from lettuces (*n* = 11), carrots (*n* = 1) and tomatoes (*n* = 1) were all susceptible to trimethoprim/sulfamethoxazole.

Overall, *E. coli* (*n* = 2), *M. morganii* (*n* = 1), *Acinetobacter* spp. (*n* = 1), *Enterobacter* spp. (*n* = 1) and *K. pneumoniae* (*n* = 1) showed multidrug resistance.

Synergy tests identified presumptive phenotypic production of ESBL, AmpC β-lactamase, metallo-β-lactamases, penicillinases, and co-production of those β-lactamases (Figure S1). Their detection was associated with the origin of the samples to determine that 60.3% and 32% of the isolates recovered from conventionally and organically produced foodstuffs, respectively, were presumptive AmpC producers; 0.8% isolates from conventionally produced items were identified as phenotypically positive for ESBL production by the presence of synergy between cefotaxime and clavulanic acid, ceftazidime, and clavulanic acid or both (Figure S1). Overall, 80.1% of the isolates were presumptive producers of penicillinases. Metallo-β-lactamases were also presumptively produced by 1.6% of the isolates, distributed among conventional and organic recovered isolates grown exclusively on the soil (Figure S1).

### Genomic characterization of integron-harboring isolates

The main statistics obtained with the *de novo* assembly of the 11 genomes are displayed in Table S3. The draft genomes varied among 3.8 Mb and 3688 protein-coding genes for *M. morganii*, and 6.5 Mb and 5938 protein-coding genes for *P. putida*, respectively (Table S3).

Overall, among the 333 isolates studied, we have detected 11 isolates harboring 12 integrons (Table 3). A single *K. pneumoniae* isolate harbored a class 1 and a class 3 integron within its genome. Integrons were similarly recovered along the whole period of sampling (March 2013 to February 2014), distributed among organic (*n* = 6) and conventional (*n* = 5) food products, and were predominant among lettuces (*n* = 9), but were also present in carrots (*n* = 1) and strawberries (*n* = 1; Table 3).

**Table 3:** **Phenotypic and genotypic characteristics of isolates producing class 1, 2, and 3 integrons**.

**Isolate**	**Sample**	**Date**	**Type of production**	**Produce**	**NS profile**	**Acquired antibiotic resistance genes**	**VF**	**Human pathogen probability (%)^a^**	**MLST**	**Plasmids**	**Number of phage regions**	**Integron classes**
***E. cloacae***												
INSali2	VG2	March 2013	Convent.	Lettuce	A, Ac, F, S	*aadA1b, bla*TEM−1, *dfrA1b, sul1, tetA*	–	75.2	ST636	IncX	2	1
INSali10	VG7	March 2013	Organic	Lettuce	A, Ac, Cr, F, G	*aadA2, aadB, qnrA1, sul1*	–	75.6	ST90	IncFIB, IncFII	2	1
***E. coli***												
INSali25	VG26	May 2013	Convent.	Lettuce	A, N, G, C	*aadA1y, aph(4)-Ia, bla*TEM−1, *estX-12, floR, mcr-1, sat2, strA, strB, sul2, tetA*	*gad*	93.4	ST1716	IncHI2, IncI1, IncI2, IncQ, IncP, IncY	6	2
INSali 38	VG37	June 2013	Convent.	Lettuce	A, G	*aac(3)-Via, aadA1a, bla*TEM−1, *strA, strB, sul1, sul2, tetA*	*lpfA, capU*	94.2	ST5981	IncHI2, IncFIC, IncP, IncQ	3	1
INSali 92	VG85	October 2013	Organic	Lettuce	A, C	*aadA1b, bla*TEM−1, *catA1, dfrA1b, strA, strB, sul1, sul2, tetA*	*gad, lpfA*,	94.2	ST2522	IncHI2, IncP, IncQ	2	1
INSali 370	VG85	October 2013	Organic	Lettuce	A, N, Ci, S	*aadA2, bla*TEM−1, *dfrA12, strA, strB, sul1, sul2, tetA*	*lpfA*	94.0	ST345	IncQ	5	1
***R. planticola***												
INSali 127	VG131	January 2014	Organic	Carrot	A	*aadA1y, sat2*	–	83.2	NA	IncL/M	4	2
INSali 133	VG128	January 2014	Organic	Lettuce	A	*aadA1y, sat2*	–	83.1	NA	IncL/M	4	2
***M. morganii***												
INSali 207	VG26	May 2014	Convent.	Lettuce	A, Ac, N, G, C	*aadA1a, aadB, cmlA1d, mphA, sul1, tetB*	–	66.6	NA	–	3	1
***P. putida***												
INSali382	VG129	January 2014	Organic	Lettuce	–	–	–	8.8	NA	–	1	1
***K. pneumoniae***												
INSLA 390	VG136	February 2014	Organic	Strawberry	A, Ac, Ct, Cr, N, Ci, S	*aadA2br, bla*GES−11, *bla*SHV−28, *dfrA12, dfrB3, oqxAB, sul1 tetD*	–	87.8	ST15	IncFIB, IncQ, IncR, IncFII	4	1, 3

a *Probability of isolate being a human pathogen according with Pathogen Finder (http://www.cge.cbs.dtu.dk/services/PathogenFinder); A, ampicillin; Ac, amoxicillin with clavulanic acid; Ct, cefotaxime; Cr, ceftazidime; F, cefoxitin; N, nalidixic acid; Ci, ciprofloxacin; G, gentamicin; C, chloramphenicol; S, trimethoprim/sulfamethoxazole; Convent., Conventional; NS, Non-susceptibility; VF, virulence factors; MLST, Multilocus sequence typing; NA, Not applicable*.

Besides the presence of typically chromosomal resistance genes such as *bla*_ACT−type_ and *bla*_DHA−type_ in *E. cloacae*, and *M. morganii* (data now shown), respectively, 10 out of the 11 isolates showed diversity of acquired resistance genes (Table 3). These genes may be able to confer resistance to multiple antibiotic classes, such as β-lactams, aminoglycosides, quinolones, chloramphenicol, and sulfonamides, as represented in the susceptibility profile displayed in Table 3. Among the acquired resistance mechanisms, we highlight the presence of the recently described plasmid-mediated colistin resistance gene *mcr-1* in *E. coli*, and the macrolide inactivation gene *mphA* in *M. morganii*, both from lettuce samples, as well as ESBL-encoding gene *bla*_GES−11_ and PMQR-encoding gene *oqxAB* in *K. pneumoniae* recovered from organic strawberries.

The assessment of STs, showed that one of the *E. cloacae* and one of the *E. coli* isolates were assigned to new allele combinations, that were registered in the respective MLST databases as ST636 and ST5981 (Table 3). In addition, other STs were detected for the remaining typable species: *E. cloacae* (ST90), *E. coli* (ST345, ST1716, and ST2522) and *K. pneumoniae* (ST15) (Table 3).

According with PlasmidFinder, the majority of the isolates harbored known *Enterobacteriaceae* plasmids from different incompatibility groups, among which we highlight the predominance of IncQ (INSali10, INSAli25, INSali38, INSali92, INSali370, and INSali390). Globally, 1–6 intact prophage regions were detected within the genomes of these Gram negative isolates. For *E. coli* isolates INSali25, INSali38, INSali92, and INSali370, virulence factors *gad, lpfA, capU* were differentially detected, as displayed in Table 3.

According with PathogenFinder, the majority of the isolates showed relevant similarity with human pathogens: the values have leveled off between 66.6% for *M. morganii* INSali207 and 94.2% for the clinically relevant *E. coli* isolates INSali38 and INSali92*. P. putida* INSali382 was the only integron-positive isolate predicted as a non-human pathogen (8.8%), showing also no acquired antibiotic resistance genes (Table 3).

### Diversity of mobile resistance integrons

Among the eight class 1 integrons analyzed, two were assigned to new integron numbers due to the detection of novel gene cassette arrays.

**Figure 2:**
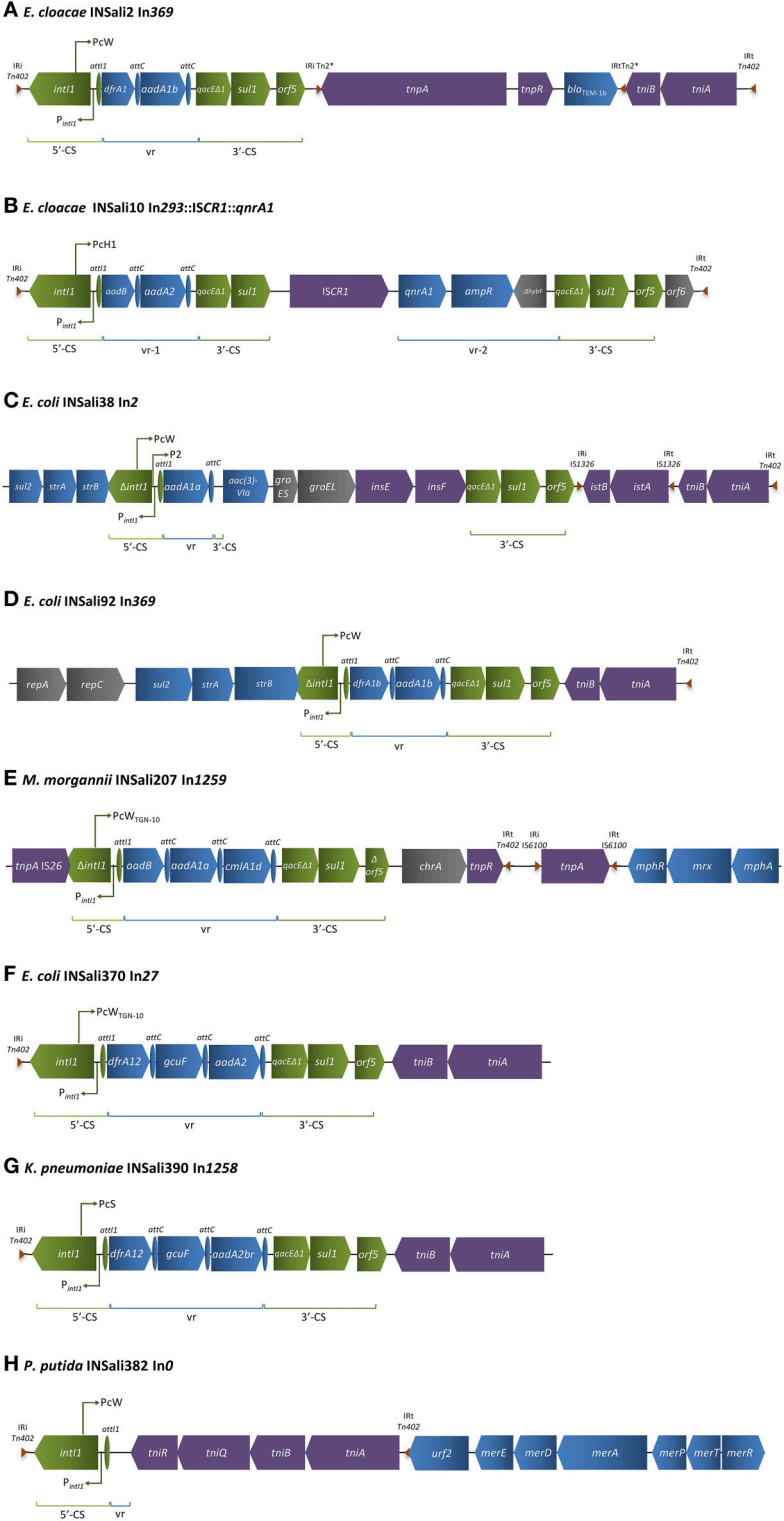
**Schematic representation of regions enclosing class 1 integrons detected among the bacterial population analyzed in the present survey (*n* = 8/333). (A)** *E cloacae* INSali2 In369; **(B)** *E. cloacae* INSali10 In*293*::IS*CR1*::*qnrA1*; **(C)** *E. coli* INSali38 In*2*; **(D)** *E. coli* INSali92 In*369*; **(E)** *M. morgannii* INSali207 In*1259*; **(F)** *E. coli* INSali370 In*27*; **(G)** *K. pneumoniae* INSali390 In*1258*; **(H)** *P. putida* INSali382 In*0*. Green, integron; blue, resistance genes, including gene cassettes; purple, transposons; gray, other genes.

Globally, an assortment of promoters, gene cassette arrays and associated transposable elements were detected. Several Tn*402*-like class 1 integrons were recovered in this study. The 12,038 bp sequence of isolate *E. cloacae* INSali2 (Figure 2A) was assigned to In*369*, and enclosed an array containing gene cassettes *drfA1b* and *aadA1b*, encoding resistance to trimethoprim and aminoglycosides, respectively. A single complex integron was recovered (In*293*::IS*CR1*::*qnrA1*) in *E. cloacae* INSali10 (Figure 2B). The integron showed a variable region 1 (vr-1) comprised of *aadB* and *aadA2* genes, and variable region 2 (vr-2) included a *qnrA1*, an *ampR* and a Δ*hybF*, which encode a zinc-containing protein associated with NiFe hydrogenase. Integron from *E. coli* INSali38 (Figure 2C) exhibited an *intI1* interrupted by the insertion of a cluster of three resistance genes (*sul2-strA-strB*). In this case, integron variable region enclosed only one gene cassette with an identifiable open reading frame and attendant *attC* site: *aadA1a*. Similarly to INSali38, 11,395 bp Tn*402*-like integron harbored by *E. coli* INSali92 was preceded by *repAC* and *sul2-strA-strB* gene cluster, interrupting *intI1* gene (Figure 2D). The gene cassette array of this In*369*, which was identical to INSali2, included *drfA1b* and *aadA1b* antibiotic resistance genes. In*1259* was firstly detected *in M. morganii* INSali207 due to the presence of a new gene cassette array (Figure 2E). It is worth noting that the downstream flanking region was composed by an IS*6100, a* chromate transporter-encoding gene *chrA*, and by macrolide inactivation gene cluster *mphA-mrx-mphR*. *E. coli* INSali370 and *K. pneumoniae* INSali390 harbored similar Tn*402*-like integrons. However, while In*27* enclosed *dfrA12, gcuF* and *aadA2* in the variable region (Figure 2F), in the new In*1258* (Figure 2G) *aadA2* was substituted by the novel derivative *aadA2br*. The class 1 integron present in the genome of *P. putida* INSali382 contained a variable region for which no significant homology could be found in existing integrons (Figure 2H). In this In*0*, gene cassettes for known antibiotic resistance determinants were not present, neither the traditional 3′CS region encoding *qac*Δ*E1, sul1* and *orf5*. This Tn*402*-like integron was limited by a complete Tn*402* transposition module – *tni*ABQR – which was followed by a *mer* operon, encoding resistance to mercury.

**Figure 3:**
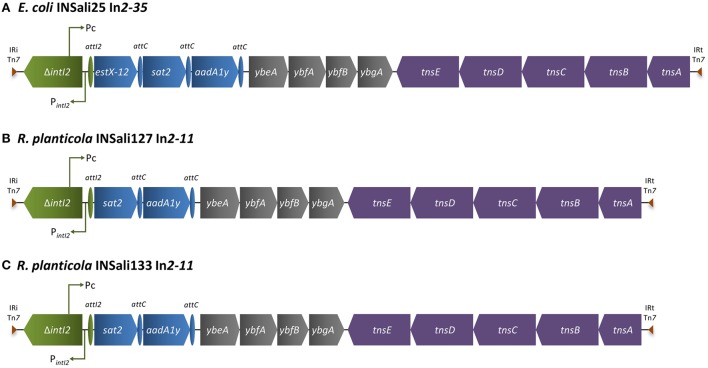
**Schematic representation of regions enclosing class 2 integrons detected among the bacterial population analyzed in the present survey (*n* = 3/333). (A)** *E. coli* INSali25 In*2–35*; **(B)** *R. planticola* INSali127 In*2–11*; **(C)** *R. planticola* INSali133 In*2–11*. Green, integron; blue, resistance genes, including gene cassettes; purple, transposons; gray, other genes.

Different class 2 integrons were detected in *E. coli* INSali25 (In*2–35*), and in *R. planticola* INSali127 and INSali133 isolates (In*2–11*) (Figures 3A–C). Just like the majority of class 2 integrons, the gene cassette arrays included genes coding for resistance to aminoglycosides and streptothricin, as follows: *estX12-sat2-aadA1y* for *E. coli* INSali25 and *sat2-aadA1y* for *R. planticola* INSali127 and INSali133 (Figures 3A–C).

**Figure 4:**
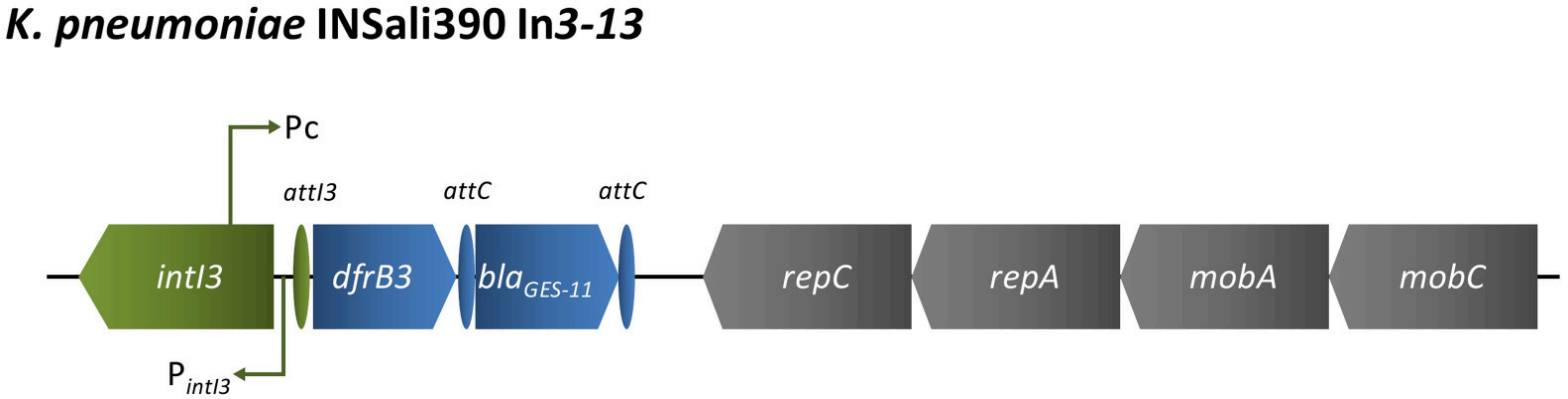
**Schematic representation of region enclosing In*3–13* detected in a *K. pneumoniae* recovered among the bacterial population analyzed in the present survey (*n* = 1/333)**. Green, integron; blue, resistance genes, including gene cassettes; purple, transposons; gray, other genes.

The new class 3 integron In*3–13* was detected in *K. pneumoniae* INSali390 isolate (Figure 4) that also carried the new In*1258* (Figure 2G). The 8,873 bp length region showed 100% identity with In*3–9*, whose sequence was recently submitted to Genbank (KT984195), after being detected in *Citrobacter freundii* from a hospital effluent in France. The variable gene array enclosed dihydrofolate reductase type B-encoding gene *dfrB3*, which was followed by the ESBL-encoding gene *bla*_GES−11_. The array of acquired antibiotic resistance genes was followed by genes encoding proteins essential for plasmid replication (*repC, repA)* and mobilization (*mobC, mobA*) (Figure 4).

## Discussion

The current study focused on evaluating organically and conventionally grown produce with regard to antibiotic non-susceptibility and integron content. Overall, it led to three main global findings. First, Gram negative bacteria were isolated from raw and unwashed fruits and vegetables. Detection of antibiotic resistant Gram negative bacteria from different species in distinct samples from retail vegetables, upon selection of different morphotypes, showed diversity of bacterial species and an assortment of antibiotic susceptibility patterns and mechanisms. In fact, colony morphotype approaches have the potential to either over or underestimate true species diversity, due to variation in colony morphology within individual species or to inability to differentiate a colony morphology common to multiple species (Lebaron et al., 1998). Thus, in this study we focused on the evaluation and assessment of diversity while any quantitative conclusions were cautiously drawn. Overall, similar species distribution was detected in vegetable products from organic and conventional production (Table S2). It should however be noted that inappropriate manipulation of fresh produce could contribute in some degree to the presence of antibiotic resistance bacteria in food products (van Hoek et al., 2015). Second, comparison with other reports of Gram negative bacteria recovered from vegetables revealed that this study found lower levels of resistance. These often included non-susceptibility to third generation cephalosporin in *Enterobacteriaceae*, suggestive of ESBL production (Kim et al., 2015). The third main conclusion was that certain organic and conventional products carried isolates harboring class 1, 2, and/or 3 integrons that also carried non-related clinically relevant acquired antibiotic resistance genes, which might contribute to the spread of antibiotic resistance in the food chain.

The bacterial genera detected in the assessed fresh produce could be traced back to different origins: although some genera such as *Delftia* spp., *Erwinia* spp., and *Comamonas* spp. are mainly environmental, the majority of the genera comprised bacteria that are often described as commensal and clinically relevant, such as *E. coli* and *K. pneumoniae* (Figure 1). In spite of this, the majority of the identified taxa (e.g., *Leclercia* spp., *Pantoeae* spp.) included bacterial genera that are usually recovered from the environment but are also able to cause infections (Guentzel, 1996; Marshall et al., 2009). For instance, many species of *Enterobacter* spp. are typically environmental. However, *Enterobacter* spp. is also often described as an intestinal commensal of humans and animals, and is frequently isolated from cases of hospital- and community-acquired urinary tract infections in immunocompetent and immunocompromised hosts (Chang et al., 2013). In this study, *E. cloacae* isolates were also among integron producers. Other important *Enterobacteriaceae* human commensals, such as *E. coli, M. morganii, Pseudomonas* spp., and particularly *Acinetobacter* spp., which are primarily pathogenic for immunocompromised hosts, were also frequently detected in this study. The conclusions about the influence of vegetables in the ecology of the human microflora are diverse. In fact, there is evidence that the prevalence of antibiotic resistant bacteria in the gastrointestinal flora may vary with dietary habits (van Den Braak et al., 2001). In Europe, reports of antibiotic resistance bacteria in food products imported from other continents is frequent (Wang et al., 2011; Zurfluh et al., 2015a,b; Zurfuh et al., 2016). However, in our study, samples were obtained from products grown and marketed in Portugal, suggesting that international food trade isn't always a requirement for contamination of fresh produce with antibiotic resistant bacteria.

The genomic characterization of isolates harboring class 1, 2, and 3 integrons showed that these genetic elements were mostly found among commensal bacteria and opportunistic pathogens. In fact, the similarity of these microorganisms with human pathogens, estimated upon the detection of γ*-proteobacteria* pathogenicity and non-pathogenicity factors, showed that only *P. putida* INSali382 was not a putative human pathogen. *R. planticola* is not often associated with human infections (Olson et al., 2013; Lam and Salit, 2014). In this study, this species was isolated from organic samples of lettuce and carrot collected at the same retail store. Indeed, the genetic similarity between isolates INSali127 and INSali133 suggests the contamination of both products by a single source. Moreover, *E. coli* isolates ST2522 INSLA92 and ST345 INSLA370 were recovered from the same lettuce sample, representing an example of contamination of a unique produce with multiple antibiotic resistance microorganisms. Moreover, multidrug resistant ST15 *K. pneumoniae*, which in this study was detected in organically-produced strawberries, represents a major clone among nosocomial infections, as previously reported (Hu et al., 2013; Markovska et al., 2015), namely in Portugal (Manageiro et al., 2015).

When we analyzed the acquired resistance mechanisms from the 11 integron-producing isolates, some specific genes hold our attention. The identification of *mcr-1* gene in an *E. coli* recovered from a conventionally produced lettuce, corroborated that the food chain may be involved in the dissemination of colistin transferable resistance genes (Hasman et al., 2015; Liu et al., 2015; Stoesser et al., 2016; Yao et al., 2016). Considering that colistin is a last resource antibiotic, used for the treatment of infections caused by multidrug resistant bacteria, the detection of a mobile colistin resistance gene in a raw vegetable constitutes a serious and unprecedented public health concern (Paterson and Harris, 2016). Although unusual, the presence of the *mphA* gene cluster in *M. morganii* is of little clinical significance, because *Enterobacteriaceae* are intrinsically resistant to macrolides due to the presence of efflux transporters (Leclercq, 2002; Poole et al., 2006). However, considering that the human gut is prone to the occurrence of increased horizontal gene transfer between bacteria, *mphA* gene might get transferred to commensal Gram positive bacteria (Stecher et al., 2012); this and the supplementary array of plasmids and prophages harbored by integrons-positive isolates reinforced the potential concerted contribution of different mobile genetic elements to the mobilization and spread of acquired resistance (Fernandez-Lopez and De La Cruz, 2014).

We choose to focus the investigation mainly on specific elements associated with the presence of acquired resistance in the food chain. Globally, the identification of class 1 (*n* = 8), class 2 (*n* = 3) and class 3 (*n* = 1) integrons has led to the identification of different gene cassettes and diversity of integrons backbones.

In this study, Tn*402*-like class 1 integrons were identified in isolates INSali2, INSali 10, INSali38, INSali92, INSali370, INSali382, and INSali390. This transposon is bound by two inverted repeats (IRi and IRt) and acts as the main carrier element for class 1 integrons, which usually display an incomplete transposition module composed of two *tni* open reading frames (Gillings et al., 2009; Sajjad et al., 2011). Notably, class 1 integrons showed an impressive mesh of interactions with several transposable elements (e.g., Tn*402, ISCR*1, Tn2^*^, IS*26*, IS*1326*, and IS*3*), which, in some cases, were flanked by regions that denote their presence in resistance plasmids (*rep* and *mob* genes). The In*0* described in isolate *P. putida* INSali382 showed unusual 3′CS context, displaying active transposition machinery, which enables greater mobility. Apart from the example reported here in bacterial population of fresh produce, other recent examples of class 1 integrons linked to functional Tn*402* modules include bacteria from other food items (Sajjad et al., 2011), clinical samples (Chen et al., 2014) and the environment (Rosewarne et al., 2010). Overall, Pc variants with different strengths were also detected in the class 1 integrons, based on the sequence of the −35 and −10 elements. From the eight variants initially described, we detected weak promoter PcW (*n* = 4), hybrid promoter PcH1 (*n* = 1), and the stronger variants PcW_TNG−10_(*n* = 2) and PcS (*n* = 1) and the additional P2 (*n* = 1). Although the predominant PcW is described as the weakest promoter of all, it is also associated with higher integrase excision activity, compensating the lower expression levels of the variable gene array with an increased ability to incorporate gene cassettes (Vinué et al., 2011).

The class 2 integrons detected in one *E. coli* and in two genetic related *R. planticola isolates* corresponded to In*2–35* and In*2–11*, already described in *E. coli* from animals and *V. cholerae* O1 clinical strain (Kadlec and Schwarz, 2008; Sá et al., 2010). As noted in this study, class 2 integrons normally show less variability than class 1 and 3 integrons, due to the lack of integration activity of *intI2* gene, which is commonly truncated. The frequent persistence of genes such as *estX* and *sat* genes in a considerable number of class 2 integrons, suggests co-selection of these genes even in the absence of a direct selective pressure. In fact, streptothricin antibiotics have not been used as therapeutics, but for a long time this antibiotic has been used as growth promoters in veterinary, which justifies their current widespread distribution (Looft et al., 2012). Overall, the gene cassettes detected within the variable region of class 2 integrons provide a smaller contribution to multidrug resistance phenotypes, than those of class 1 integrons (Ramírez et al., 2010).

The variable region of the new class 3 integron (In*3–13*) detected in *K. pneumoniae* INSali390 exhibited a *dfrB3* and a *bla*_GES−11_. GES-containing class 3 integrons have already been described. Curiously, two of them, In*3*−*2* [*bla*_GES−1_, *bla*_OXA−10_/*aac(6*′*)-Ib*] and In*3–8* (*bla*_IMP−8_,*bla*_GES−5_, *bla*_BEL−1_, *aacA4*), were described in *K. pneumoniae* from Portuguese health care settings in 2003 and 2015, respectively (Correia et al., 2003; Papagiannitsis et al., 2015). Considering this information, the report of a GES-containing class 3 integron in a pathogenic *K. pneumoniae* suggests food contamination from clinical sources. At the boundaries of the In*3-13* we detected genes associated with plasmid replication (*repA, repC*) and mobility (*mobA, mobC*), confirming its transference potential. This corroborated results from other class 3 integron-containing plasmids that have showed the presence of *rep* genes downstream of the gene cassettes (Papagiannitsis et al., 2015). Although class 3 integrons have been associated with clinical and environmental samples, to the best of our knowledge, this study constitutes the first assigned class 3 integron from food products of vegetable origin (http://integrall.bio.ua.pt/). Overall, the pathogenic ST15 *K. pneumoniae* producer of new In*1258* and In*3-13* recovered from organic strawberries constitute an additional a food safety concern.

## Conclusion

This study confirms fresh produce as a relevant reservoir of Gram negative bacteria carrying antibiotic resistance determinants that are widespread in clinical settings. However, it is evidenced that the vegetable Gram negative resistome in Portugal is not significantly influenced by the type of agricultural production. The detection of integrons of three different classes, associated with clinically relevant mobile genetic elements and acquired antibiotic resistance genes, such as *mcr-1*, reinforce the mobilization potential of antibiotic resistance in Gram negative bacteria. Although continuous monitoring of products of vegetable and animal origin is essential, the decrease or cessation of certain antibiotics in manure and agriculture is strongly advised to preserve drug molecules for clinical treatment of infections.

## Author contributions

DJ designed the study, acquired laboratory and epidemiological data, analyzed the data and wrote the manuscript. VM analyzed the data and reviewed the manuscript. EF acquired laboratory data. PB acquired laboratory data. LV acquired laboratory data. IM acquired laboratory data and reviewed the manuscript. MC designed the study and reviewed the manuscript. All authors read and approved the final manuscript.

## Funding

DJ has received research funding from Fundação para a Ciência e a Tecnologia (FCT, grant number SFRH/BD/80001/2011). VM was supported by FCT fellowship (grant SFRH/ BPD/77486/2011), financed by the European Social Funds (COMPETE-FEDER) and national funds of the Portuguese Ministry of Education and Science (POPH-QREN). We thank the support of FCT grant number PEst-OE/AGR/UI0211/2011-2014 and UID/MULTI/00211/2013.

### Conflict of interest statement

The authors declare that the research was conducted in the absence of any commercial or financial relationships that could be construed as a potential conflict of interest.

## Supplementary material

Figure S1 **Percentage of Gram negative bacteria according with the mode of production and level of growth of fresh produce, taxonomic group and phenotype indicative of penicillinases, AmpC β-lactamase, extended-spectrum β-lactamase (ESBL), and metallo-β-lactamase (MBL) production (*n* = 333)**.

Table S1 **Distribution of 333 isolates according with date of mode of fresh produce production, type of growth and antibiotic selection**.

Table S2 **Distribution of 333 isolates according with bacterial genera, mode of production and level of growth**.

Table S3 **Genome analysis of Gram negative isolates carrying integrons**.
