# Supplementary figures and images for: Architecture of Class 1, 2, and 3 Integrons from Gram Negative Bacteria Recovered among Fruits and Vegetables
Source: Front Microbiol. 2016 Sep 13;7:1400. doi: 10.3389/fmicb.2016.01400 (PMC5020092; doi:10.3389/fmicb.2016.01400)

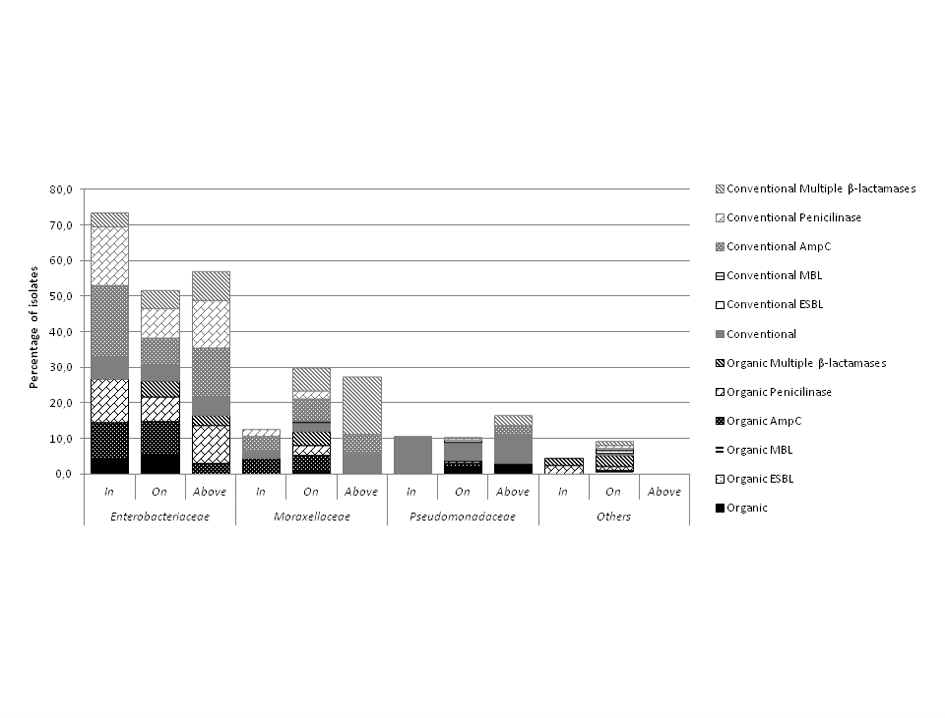

Supplement: Figure S1 — Percentage of Gram negative bacteria according with the mode of production and level of growth of fresh produce, taxonomic group and phenotype indicative of penicillinases, AmpC β-lactamase, extended-spectrum β-lactamase (ESBL), and metallo-β-lactamase (MBL) production (n = 333). [file Image1.TIF]
