# Supplementary material for: Architecture of Class 1, 2, and 3 Integrons from Gram Negative Bacteria Recovered among Fruits and Vegetables
Source: Front Microbiol. 2016 Sep 13;7:1400. doi: 10.3389/fmicb.2016.01400 (PMC5020092; doi:10.3389/fmicb.2016.01400)
Supplement: Table S1 — Distribution of 333 isolates according with date of mode of fresh produce production, type of growth and antibiotic selection. [file Table1.DOCX]

| **Antibiotic** | **Organic** | | | **Conventional** | | |
| --- | --- | --- | --- | --- | --- | --- |
|  | **In** | **On** | **Above** | **In** | **On** | **Above** |
| **Amoxicillin** | 13 | 45 | 6 | 17 | 35 | 10 |
| **Cefotaxime** | 1 | 10 | 0 | 2 | 16 | 6 |
| **Ceftazime** | 0 | 12 | 0 | 4 | 15 | 3 |
| **Ertapenem** | 11 | 1 | 1 | 18 | 3 | 5 |
| **Imipenem** | 0 | 3 | 0 | 0 | 6 | 0 |
| **Tetracycline** | 2 | 14 | 0 | 4 | 16 | 1 |
| **Chloramphenicol** | 0 | 9 | 0 | 1 | 10 | 5 |
| **Nalidixic acid** | 0 | 4 | 0 | 1 | 7 | 0 |
| **Ciprofloxacin** | 0 | 1 | 0 | 0 | 1 | 0 |
| **Gentamicin** | 0 | 5 | 0 | 0 | 9 | 0 |

**Table S1**. Distribution of 333 isolates according with date of mode of fresh produce production, type of growth and antibiotic selection.
