# Supplementary material for: Architecture of Class 1, 2, and 3 Integrons from Gram Negative Bacteria Recovered among Fruits and Vegetables
Source: Front Microbiol. 2016 Sep 13;7:1400. doi: 10.3389/fmicb.2016.01400 (PMC5020092; doi:10.3389/fmicb.2016.01400)
Supplement: Table S2 — Distribution of 333 isolates according with bacterial genera, mode of production and level of growth. [file Table2.DOCX]

**Table S2**. Distribution of 333 isolates according with bacterial genera, mode of production and level of growth.

| **Genera** | **Mode of production** | **Level of growth** | | |
| --- | --- | --- | --- | --- |
|  |  | **In the soil** | **On the soil** | **Above the soil** |
| *Acinetobacter* | Organic | 2 | 29 | 0 |
|  | Conventional | 4 | 44 | 10 |
| *Aeromonas* | Organic | 1 | 2 | 0 |
|  | Conventional | 0 | 3 | 0 |
| *Cedecea* | Organic | 0 | 0 | 0 |
|  | Conventional | 0 | 1 | 0 |
| *Citrobacter* | Organic | 2 | 2 | 0 |
|  | Conventional | 0 | 0 | 1 |
| *Comamonas* | Organic | 0 | 2 | 0 |
|  | Conventional | 0 | 1 | 0 |
| *Delftia* | Organic | 0 | 1 | 0 |
|  | Conventional | 0 | 2 | 0 |
| *Enterobacter* | Organic | 4 | 33 | 2 |
|  | Conventional | 9 | 23 | 7 |
| *Erwinia* | Organic | 0 | 0 | 0 |
|  | Conventional | 0 | 1 | 0 |
| *Escherichia* | Organic | 0 | 3 | 0 |
|  | Conventional | 0 | 7 | 0 |
| *Klebsiella* | Organic | 1 | 6 | 3 |
|  | Conventional | 2 | 2 | 1 |
| *Leclercia* | Organic | 0 | 0 | 0 |
|  | Conventional | 1 | 0 | 0 |
| *Morganella* | Organic | 0 | 1 | 0 |
|  | Conventional | 0 | 7 | 0 |
| *Pantoea* | Organic | 0 | 1 | 0 |
|  | Conventional | 0 | 0 | 1 |
| *Proteus* | Organic | 0 | 0 | 0 |
|  | Conventional | 0 | 1 | 0 |
| *Providencia* | Organic | 0 | 1 | 0 |
|  | Conventional | 0 | 4 | 0 |
| *Pseudomonas* | Organic | 0 | 8 | 1 |
|  | Conventional | 5 | 16 | 5 |
| *Rahnella* | Organic | 4 | 6 | 1 |
|  | Conventional | 1 | 0 | 3 |
| *Raoutella* | Organic | 1 | 5 | 0 |
|  | Conventional | 4 | 7 | 1 |
| *Serratia* | Organic | 1 | 6 | 0 |
|  | Conventional | 6 | 10 | 1 |
| *Stenotrophomonas* | Organic | 1 | 8 | 0 |
|  | Conventional | 0 | 4 | 0 |
