# Supplementary material for: Architecture of Class 1, 2, and 3 Integrons from Gram Negative Bacteria Recovered among Fruits and Vegetables
Source: Front Microbiol. 2016 Sep 13;7:1400. doi: 10.3389/fmicb.2016.01400 (PMC5020092; doi:10.3389/fmicb.2016.01400)
Supplement: Table S3 — Genome analysis of Gram negative isolates carrying integrons. [file Table3.DOCX]

| **Isolates** | **Genome size (bp)** | **Number of contigs** | **Average coverage** | **Integron contig** | **N50 (bp)** | **Maximum contig (bp)** | **Minimum contig (bp)** | **Protein-coding genes** | **RNAs** |
| --- | --- | --- | --- | --- | --- | --- | --- | --- | --- |
| **INSAli2** | 4,793,038 | 42 | 145.7 | 5 | 344,398 | 466,839 | 597 | 4,471 | 76 |
| **INSAli10** | 4,949,513 | 63 | 113.2 | 15 | 251,933 | 681,923 | 323 | 4,618 | 80 |
| **INSAli25** | 5,249,548 | 165 | 140.9 | 35 | 99,794 | 250,299 | 403 | 5,254 | 82 |
| **INSAli38** | 5,057,715 | 107 | 149.2 | 29 | 128,829 | 535,543 | 347 | 4,904 | 82 |
| **INSAli92** | 4,905,451 | 87 | 174.9 | 41 | 167,610 | 450,780 | 401 | 4,686 | 77 |
| **INSAli127** | 6,011,051 | 81 | 107.7 | 6 | 185,876 | 476,313 | 407 | 5,731 | 77 |
| **INSAli133** | 6,011,856 | 77 | 110.4 | 10 | 156,558 | 496,480 | 512 | 5,729 | 79 |
| **INSAli207** | 3,806,004 | 79 | 151.2 | 32 | 213,305 | 315,226 | 422 | 3,688 | 78 |
| **INSAli370** | 4,743,192 | 110 | 132.0 | 37 | 131,062 | 407,142 | 403 | 4,559 | 68 |
| **INSAli382** | 6,490,961 | 246 | 51.3 | 101 | 53,313 | 230,406 | 373 | 5,938 | 66 |
| **INSAli390** | 5,566,516 | 88 | 130.1 | 52 (*intI1*);  27 (*intI3*) | 177,301 | 429,033 | 417 | 5,240 | 82 |

**Table S3.** Genome analysis of Gram negative isolates carrying integrons.
